# Supplementary material for: Are carbon emissions trading and green financial instruments synergistic? -Comprehensive quantitative research based on content analysis
Source: PLoS One. 2024 Mar 7;19(3):e0298601. doi: 10.1371/journal.pone.0298601 (PMC10919601; doi:10.1371/journal.pone.0298601)
Supplement: S1 Data — (ZIP) [file pone.0298601.s001.zip › Data/地级市Z_F工作报告.zip!/▒▒╛⌐/└┤╫╘╣1⁄2╓┌║┼í╛┬φ┐╦╩2╛▌═°í┐_2004.docx]

北京市第十二届人大第二次会议今天上午9时开幕，代市长王岐山作了政府工作报告。以下为报告全文。

　　各位代表，我代表北京市人民政府向大会作政府工作报告，请予审议，并请市政协各位委员提出意见。

　　一、2003年工作的回顾

　　2003年是首都改革开放和现代化建设进程中很不平凡的一年，是全市各族人民经受严峻考验并取得较大成绩的一年。在中共北京市委的领导下，首都人民坚持以邓小平理论和“三个代表”重要思想为指导，坚决贯彻党中央、国务院的各项方针政策和对北京市工作的一系列指示精神。以实现“新北京、新奥运”战略构想为目标，以加快发展为总基调，以优化发展环境为着力点，万众一心，奋力拼搏，扎扎实实地推进各项工作，夺取了防治非典的阶段性重大胜利，保持了经济社会全面发展的良好局面，较好地完成了市十二届人大一次会议确定的各项任务。

　　坚持“两手抓”，取得“双胜利”。2003年初，全市经济社会发展的开局很好。正当我们全面推进首都现代化建设的时候，发生了一场突如其来的非典型肺炎疫情。防治非典初期，工作一度被动。4月17日，中央果断决策，决定成立北京防治非典型肺炎联合工作小组，统一指挥北京地区非典防治工作。胡锦涛总书记多次就北京市防治非典工作作出重要指示，温家宝总理先后六次亲临北京防治非典一线检查指导工作。

　　按照中央的决策和部署，落实早发现、早报告、早隔离、早治疗的方针，围绕提高收治率和治愈率、降低病死率和医护人员感染率，我们果断采取了一系列重大措施，依靠首都和部队医务人员舍生忘死的工作，广大市民的共同奋斗，全国人民的大力支持，在两个月之内有效控制了非典疫情，实现了“双解除”。

　　与此同时，坚决贯彻“一手抓防治非典这件大事不放松，一手抓经济建设这个中心不动摇”的方针，坚持“外塑形象、内聚人心”，及时制定实施针对性措施，千方百计促进经济增长，赢得了“防非典、促发展”的双胜利。首都经济保持良好发展势头。北京市生产总值完成3611.9亿元，比上年增长10.5%。积极推进经济结构调整，以汽车制造为代表的现代制造业异军突起，高新技术产业稳步回升，工业成为拉动经济增长的重要力量，对经济增长的贡献率达到33.2%。第三产业平稳增长。

　　现代都市农业持续发展。完成全社会固定资产投资2157.1亿元，增长18.9%。实现社会消费品零售额1916.7亿元，增长14.5%。地方企业出口73.7亿美元，增长24.9%；实际利用外商直接投资21.5亿美元，增长19.8%。经济运行的质量和效益进一步提高，全市规模以上工业企业实现利润219.5亿元，增长40.6%；地方财政收入592.5亿元，增长18.2%。

　　人民生活水平不断提高，城镇居民人均可支配收入13882.6元，实际增长11.2%；农民人均纯收入6496.3元，实际增长11.5%；年人均纯收入2500元以下的低收入村已经消除。城乡社会救助工作取得成效，社会保障体系不断完善。就业形势保持稳定，城镇登记失业率为1.43%，失业人员就业率达到68.1%。

　　各项重大改革稳步推进。市政府机构改革顺利完成。议事协调机构和临时机构由97个减少到48个，精简比例达到50.5%。推进政府职能转变，取消56项行政审批事项，总结推广全程办事代理制，发展环境得到改善。国有资产管理体制改革迈出新的步伐，国有企业改革继续推进。

　　非公有制经济健康发展。制定实施《城市基础设施特许经营办法》，投融资体制改革取得突破。中关村科技园区管理体制进一步完善。企业信用建设取得新的进展。农村税费改革全面推开。整顿和规范市场经济秩序取得阶段性成果。

　　城市建设和管理水平进一步提高。以交通为重点的城市基础设施建设步伐加快。地铁五号线建设全面铺开，八通线通车试运营，实现了轨道交通竣工通车40公里的目标。城市快速道路系统建设力度加大，五环路全线贯通。南水北调工程北京段顺利开工，转河和坝河得到治理。完成了北京城市空间发展战略研究工作。

　　调整了危旧房改造的思路和模式，加强了历史文化名城保护，明十三陵列入世界文化遗产，元大都土城遗址公园建成开放。下大气力治理城乡环境卫生，完成158项环境整治重点项目，环境总体水平又有新的提高。采取更加严格的措施治理大气污染，市区空气质量二级和好于二级的天数达到61%。

　　城近郊区污水处理率实现56%，生活垃圾无害化处理率达到91.3%。新增城区大型绿地213公顷，为前四年的总和。城近郊区绿化覆盖率达到41%，全市林木覆盖率达到47.5%。城市管理重心下移取得进展，行政处罚权相对集中，综合执法得到加强。奥运会筹备工作顺利推进，发布奥运会徽，启动市场开发计划，国家体育场、国家游泳中心等重点场馆如期开工。60件实事全面落实。

　　各项社会事业不断进步。经过抗击非典斗争，更加重视经济社会协调发展。建立了可持续控制非典的工作机制。加强公共卫生基础设施的投入和建设，以大病统筹为主要形式的新型农村合作医疗制度试点顺利推进。

　　“首都二四八重大创新工程”取得新的进展，企业孵化体系建设成效显著，创新服务体系初步建立。市属科研院所体制改革进一步深化，与中央在京科技力量的合作更加紧密。中小学布局调整加快，农村普通中学规范化建设全面完成，基础教育均衡化取得进展，高等教育整体水平有所提高，教育乱收费治理取得一定成效。

　　文学艺术、新闻出版、广播影视和哲学社会科学事业不断进步，一批文化创作精品在全国获奖，文化市场繁荣、健康、有序，竞技体育发展势头良好，群众文化体育活动蓬勃开展。

　　首都精神文明建设成绩显著。在党的十六大精神鼓舞下，全市兴起学习贯彻“三个代表”重要思想新高潮，有力地促进了经济发展和社会进步。广泛开展了公民道德实践年活动。在创建文明区县活动带动下，群众性精神文明建设向广度和深度发展，社会陋习得到不同程度的改变，市民文明素质和城市文明程度有所提高。

　　深入开展“双拥”共建活动，国防教育和国防后备力量建设取得新的进展，征兵工作和优抚安置政策得到较好落实，驻京部队在首都现代化建设中发挥了重要作用。老龄工作得到加强，妇女儿童工作稳步推进，残疾人事业加快发展。

　　民主法制建设进一步加强。人大及其常委会决议得到认真执行，重大问题通过政协广泛征求意见，自觉接受法律监督和民主监督，充分听取人民群众对政府工作的意见和建议。办复全国和市人大代表议案和建议1702件，政协委员提案1229件。社区居委会换届选举顺利完成。民族、宗教、侨务和对台工作取得新进展。坚持依法治市，政府法制建设进一步加强，全市提请市人大常委会审议通过地方性法规9项，颁布政府规章31项。

　　深入开展“四五”法制宣传教育，积极拓展和规范法律服务。实施“阳光工程”，廉政建设得到加强。全面落实社会治安综合治理的各项措施，深入开展“严打”整治斗争，保持了治安形势的总体平稳。加大人民内部矛盾纠纷的排查调处力度，逐步完善维护稳定的长效机制，研究制定突发事件应急预案，全力维护首都的安全稳定。

　　各位代表，过去的一年，我们在发展中遇到的困难比预料的大，取得的成绩比预料的好，成绩来之不易。这是党中央、国务院和中共北京市委正确领导的结果，是全市各族人民团结奋斗，开拓进取的结果。

　　在此，我代表市政府，向在各个领域和岗位上辛勤劳动、作出贡献的全市人民，向给予我们支持与监督的人大代表、政协委员、各民主党派、工商联和无党派人士、各人民团体、社会各界人士，向大力支持我们工作的中央在京单位、人民解放军和武警部队以及各兄弟省、自治区、直辖市，向所有关心支持首都建设的香港特别行政区同胞、澳门特别行政区同胞、台湾同胞、海外侨胞和国际友人，表示崇高的敬意和衷心的感谢！

　　回顾过去的一年，我们清醒地看到，全市经济社会发展和政府工作中还存在着不少困难和问题：一是经济体制改革相对滞后，市场化程度较低；二是经济增长方式转变比较缓慢，增长速度与结构、质量、效益不够协调；三是产业发展的结构性矛盾仍然比较突出，尚未形成多支柱支撑体系；四是远郊区县与市区差距明显，城市化进程亟需加快，农业产业化和技术进步、农村基础设施建设和社会发展、农民的富裕和文明任重道远；五是水和土地等资源约束加剧，节约保护与合理利用工作亟待加强；六是城市建设与公共管理方面存在着一些突出矛盾和薄弱环节，交通拥堵加重，公共卫生体系建设滞后，突发事件应急预案的落实和执行不到位成为重大安全隐患；七是市民文明素质、城市文明程度需要提高，公众的法律意识、遵守公共道德和公共秩序的意识需要增强；八是政府转变职能和改进工作作风、提高办事效率的任务艰巨，电子政务推进缓慢。对上述问题，我们将高度重视，立足长远，着眼当前，统筹考虑，通过发展和改革逐步加以解决。

　　二、2004年政府工作的总体要求和主要目标

　　2004年是新中国成立55周年，是全面落实党的十六大和十六届三中全会精神的重要一年，是实现“十五”计划目标的关键一年。当前，我国正处于重要的战略机遇期，全国经济处在发展周期的上升阶段；中共中央作出《关于完善社会主义市场经济体制若干问题的决定》，为进一步解放和发展生产力指明方向；进入筹办奥运会的全面建设阶段，为首都扩大开放加快发展提供了前所未有的历史机遇。

　　当然，应当看到，国际国内经济发展仍然存在一些不确定因素，我们自身还有不少深层次问题亟待解决，首都现代化建设面临着众多挑战。我们必须切实增强责任意识、机遇意识和忧患意识，紧紧抓住、充分利用当前的大好机遇，切实保护好、引导好、发挥好各方面的积极性，全面履行“四个服务”职责，努力使首都的工作走在全国前列。

　　今年政府工作的总体要求是：以邓小平理论和“三个代表”重要思想为指导，认真贯彻党的十六大、十六届三中全会精神和中央对北京市工作的一系列重要指示，坚持以人为本，树立全面、协调、可持续的科学发展观，弘扬求真务实的精神，紧紧围绕“新北京、新奥运”战略构想，以创新体制、调整结构、优化环境、全面发展为主题，加快推进各项改革，大力发展首都经济切实加强城市建设和管理，不断推进精神文明建设、民主法制建设，实现首都经济持续快速协调健康发展和社会全面进步。

　　经济和社会发展的主要预期目标是：北京市生产总值增长9%，地方财政收入增长13%；城镇居民人均可支配收入和农民人均纯收入分别增长6%以上；城镇登记失业人员就业率保持在60%以上，城镇登记失业率控制在2.5%以内；居民消费价格指数为102%左右；人口自然增长率控制在1‰以内。

　　实现上述预期目标，要认真按照“五个统筹”、“五个坚持”的要求，从首都实际出发，科学把握改革发展的正确方向。

　　第一，必须切实推动经济增长方式的转变，努力实现速度与结构、质量、效益相统一。加快发展，不等于单纯追求经济高增长。要由主要依靠外延式发展向内涵式发展转变，由粗放经营方式向集约经营方式转变，加快信息化带动工业化的进程。紧紧抓住结构调整这条主线，着力解决投资、产业、消费等方面的结构性问题。

　　特别是要充分发挥首都人才、教育、科技、文化、卫生、体育等方面的优势，努力把资源优势转化为产业优势，在实践中不断丰富和深化首都经济的内涵。

　　第二，必须坚持社会主义市场经济的改革方向，加快改革步伐，优化发展环境，为首都经济社会发展提供强大动力。要着力推进体制创新、组织创新和制度创新，全面履行政府经济调节、市场监管、社会管理和公共服务职能，搭建符合市场经济规则的发展平台。调整不合理的利益格局，打破垄断，增强市场在资源配置中的基础性作用，使首都特有的经济决策、信息平台强势得到充分发挥。

　　第三，必须统筹城乡、区域和经济社会协调发展，促进人与自然的和谐。离开农民的小康就没有全市的小康，离开郊区的现代化就没有首都的现代化。要打破城乡分割的二元结构，引导要素资源向郊区倾斜，形成合理的产业布局，加快工业化、城市化、现代化进程。更加重视区域协调发展，促进南城与北城、城区与郊区、平原与山区、北京与周边的良性互动。

　　更加重视社会发展，推动各项社会事业全面进步。更加重视可持续发展，把控制人口规模、节约资源和保护环境摆在突出位置，努力实现经济效益、社会效益、环境效益相统一。

　　第四，必须坚持城市建设和管理并重，不断提高城市现代化管理水平。管理是政府工作的永恒主题。要坚持以规划为龙头，抓住城市空间布局战略调整和筹办奥运会的机遇，综合考虑城市功能布局，拓展城市发展空间，保护历史文化名城，为根本解决城市发展中的难点问题奠定基础。

　　大力推进城市管理体制的改革创新，建立长效机制，在规划、交通和环境卫生管理等方面实现新的突破。要把应急管理建立在完善的日常管理基础之上，不断提高城市管理和公共服务的水平。

　　第五，必须全力实现好、维护好、发展好人民群众的根本利益。牢固树立“群众利益无小事”的观念，把群众呼声和意愿作为指导工作的第一信号，把关心和服务群众作为第一职责，把群众的评价作为衡量工作政绩的第一尺度，统筹兼顾，正确处理局部利益和整体利益、眼前利益和长远利益的关系。在推进城市化过程中，维护好农民利益；

　　在推进企业改革过程中，维护好职工利益；在推进城市建设过程中，维护好居民利益；关注照顾困难群体，着力解决涉及群众切身利益的突出问题，使广大群众在改革与发展中得到更多的实惠。

　　三、全面开创首都改革开放和现代化建设的新局面。按照今年经济和社会发展的总体要求，着重做好以下五个方面的工作。(一)优化产业结构，转变经济增长方式，促进首都经济持续快速协调健康发展。根据首都经济发展的内在要求，坚持把经济结构的战略性调整作为主线，加快产业结构优化升级，夯实经济增长的基础，扩大经济总量，提高增长质量。

　　把促进消费作为扩大内需的关键环节，着力改善投资结构，实现消费和投资的“双拉动”。要充分发挥北京消费市场的优势，加快消费结构升级，增强城乡居民消费能力，扩大外来消费规模，促进消费总量扩张。引导企业围绕汽车、建材家居、数字化商品等消费热点，提高供给能力和服务质量。完善社区商业服务体系，开拓农村消费市场，倡导居民扩大旅游、体育健身和文化消费。

　　搞好假日市场。密切关注粮油、燃料等重要商品供需变化情况，加强市场监测体系和应急体系建设，提高政府调控能力。继续发挥投资对经济增长的拉动作用，进一步增强投资的产业和区域导向，从主要集中在房地产领域，向高新技术产业、现代制造业和现代服务业等有潜力的领域转移；从过于集中在市中心区，向卫星城和中心镇转移。

　　不断扩大第三产业规模，加快服务业发展，全力提升质量和水平。充分利用首都财政金融等经济决策和信息平台的独特优势，发挥商务中心区、金融街的聚集效应和服务功能，吸引国内外企业和各类金融、服务贸易机构来京投资发展。推进市属金融资产的优化配置和金融企业改革，发展多种金融服务，探索农村信用社改革，巩固和提升金融服务贸易业的强势地位。

　　发展新型商业业态，推进区域商业中心建设，优化大型购物中心布局，建设好商业、服务业特色街区。加快物流园区建设和招商引资工作，引进和培育现代化的第三方物流企业，使物流园区初具规模。巩固和拓展海外旅游市场，整合旅游资源，积极开发多档次的旅游产品，全面提升旅游会展行业素质和市场竞争力。

　　将公益性文化事业与经营性文化产业适度剥离，完善文化产业政策，打破行业垄断，吸引民间资本投入，加强文化市场培育，将文化产业发展成为首都经济新的支柱。抓住奥运机遇，引导和促进体育产业发展壮大。

　　增强高新技术产业的先导地位，强化现代制造业的支撑作用。继续发挥中关村科技园区的带动作用，加快实施《五年上台阶行动纲要》，使之成为制度创新、组织创新、技术创新新的综合改革试验区。深入推进“首都二四八重大创新工程”，支持大企业带动中小企业开展专业配套的研发活动，深化科研院所企业化转制，促使企业成为创新主体，形成一批拥有核心技术与自主知识产权的知名产品。

　　保持软件产业的领先地位，搞好软件产业基地的建设与招商工作。加快振兴现代制造业，支持汽车、微电子、光机电一体化、生物工程和新医药等产业发展，尽快形成多支柱的制造业集群。推动高新技术产业、现代制造业向郊区转移，合理布局，适当集中。积极稳妥地推进传统产业改造调整，扶植发展符合首都特点的都市型工业。全面启动奥运经济促进工作，抓紧建立相关信息服务平台，努力发展适应奥运需求的重点行业和重点产品。

　　坚持“多予、少取、放活”的方针，加快农村经济发展，促进农民增收。全面贯彻中共中央2004年一号文件精神，进一步解决好“三农”问题。深化农业结构调整，加大农业基本建设投入，落实现代农业“221行动计划”，推动特色农产品生产向优势产区集中。

　　积极发展农产品加工业，促进农业科技进步和推广，加强标准化生产和安全食品体系建设，实施产业化经营。积极发展农民专业合作经济组织。加强农村基础设施建设，完成215个行政村的通油路任务。

　　推进乡镇企业结构调整和体制创新，大力发展农村二、三产业。加快实施山区移民搬迁工程，易地安置采空区和泥石流易发区的险村险户。完善农村土地制度，保障农民对土地承包经营的权利，稳步推进农用地承包经营权流转，促进规模经营。

　　积极推进集体建设用地使用权流转试点。完善土地征用办法、补偿标准和补偿机制，健全征地与农转非挂钩的制度，维护其应享有的社会保障权利。推进农村集体资产产权制度改革，增强农村经济活力。深化农村税费改革，免征农业税，巩固农民收入增长的良好势头。

　　充分利用两个市场、两种资源，在更大范围、更宽领域、更高层次上参与国际经济技术合作与竞争。建立符合国际惯例的投资促进体系和管理服务体系，积极扩大对内对外开放，努力把北京建成最具开放性的城市。加快经济技术开发区建设，提高出口加工区管理水平，搞好对外商投资企业的服务。积极吸引国际资本、民间资本以及先进技术和高素质人才，壮大高新技术产业和现代制造业。

　　完善企业并购法规和操作办法，为中外投资者参与城市基础设施建设和国企改组改造创造条件。广泛吸引跨国公司、国内外金融机构、大企业、大集团来京设立总部、研发中心、营运中心、采购中心，发展总部经济。推进金融、商业、旅游、会展、物流等现代服务业和医疗、教育、文化等重点领域的对外开放，组织实施进一步促进京港更紧密经贸合作的若干措施。

　　深化外贸体制改革，建立健全内外贸统一的管理机制和贸易促进服务体系，使之互相促进、协调发展。积极应对出口退税政策调整对外贸出口的影响，巩固和提高“大通关”成果，提高加工贸易出口的便利化程度，采取有效措施，确保出口稳步增长。继续搞好“科博会”等大型经贸活动。做好加入世贸组织过渡期的各项应对工作。落实境外投资项目贷款贴息等优惠政策，鼓励和支持有条件的企业向海外发展。

　　加强同周边省区市的交流与合作，促进环渤海地区经济协调发展，积极参与振兴东北地区等老工业基地和西部大开发。牢牢把握首都现代化建设的关键，全面落实人才强国战略。围绕培养、吸引、用好人才，完善人才政策体系，加快建立首都人才管理体制。

　　着眼于人才总量的增长和素质的提高，加强人才资源能力建设，加大人力资本投资力度。开放首都人才市场，探索完善工作居住证等多种形式的人才引进制度。建立健全人才市场体系和服务体系，统筹人才资源整体开发，优化人才资源配置，实现人尽其才。

　　(二)统筹兼顾，突出重点，在经济体制改革方面取得新的突破。贯彻落实中央和市委关于完善社会主义市场经济体制的决定及意见，加快改革进程，为首都经济社会发展提供强有力的体制保障。深入推进行政管理体制改革，坚持不懈地优化发展环境。

　　加快政府职能转变，真正将工作重心逐步转到制定规则和标准，强化管理，加强监督和为市场主体提供优质服务上来，进一步推动政企分开、政事分开、政社分开，使政府各职能部门与所办企业和营利性事业单位彻底脱钩。

　　深化行政审批制度改革，大幅度减少年检事项，继续推广全程办事代理制。创新工作机制，严格实行工作无缺位、主办负责和投诉举报等制度，完善公务员激励和监督机制。改革政府绩效考核制度，把政府部门的工作作风、服务态度、服务质量和服务效率作为督查考核的重要内容。推进电子政务建设，加大政务公开力度。清理解决严重影响发展环境的遗留问题。循序渐进地推动事业单位改革。

　　不断完善土地、技术、劳动力等要素市场，大力发展资本市场，重点规范发展产权交易市场，促进生产要素自由流动。完善企业信用服务体系，加快建设个人信用服务体系。培育、规范各类行业协会和社会中介机构。加强知识产权保护，依法惩处盗版侵权行为。整顿和规范市场经济秩序，重点抓好食品、药品、家居建材等方面的专项整治，健全产品质量监管机制，严厉打击制假售假行为。强化旅游、文化市场监管。

　　加快投融资体制改革步伐。按照“增量改革、存量试点”的原则，开放污水垃圾处理、高速公路和轨道交通等市政公用事业和城市基础设施建设运营市场，打通社会资本进入通道。落实特许经营办法，完善投资回报补偿机制和基础设施产品定价制度，保护投资者合法权益。对经营性城市基础设施中由政府投资形成的经营权、股权，进行出让、转让。

　　转变投资管理机制，除少数重大项目和限制类项目实行核准制外，对国家没有特殊规定的企业投资建设项目实行登记备案制，对非经营性政府投资项目试行“代建制”；除市政府投资和国家规定需市政府审批的项目外，审批权下放区县。转变投资调控方式，强化产业政策引导和信息披露，搞好项目储备、推介和协调服务，防止盲目投资和重复建设。

　　转变政府投入形式和项目管理模式，建立政府投资监管和评价体系，增强政府投资的引导和放大作用，提高资金使用效益。深化财政管理改革，健全公共财政体系。转变政府资金投向，主要用于公益性和公共基础设施建设、生态环境保护等市场不能有效配置资源的领域，以及对经济结构调整有促进作用的方面。通过专项转移支付，支持南城、郊区和山区发展。

　　大力发展非公有制经济，积极推进国有资产管理体制和国有企业改革。加快清理和修订限制非公有制经济发展的规定和政策，进一步放宽市场准入，允许非公有资本进入法律法规未禁入的行业和领域，提高非公有制经济在制造业、基础设施和公共事业等领域的比重。

　　建立健全投资服务体系，完善税收政策，发展适应中小企业需要的多层次金融服务。依法保护私有财产权。提倡具有一定规模的民营企业建立现代企业制度。更多地吸引非国有资本参与国有企业的股份制改造，实现投资主体多元化。深化国有资产管理体制改革，进一步规范和完善国有资产授权经营制度。按照现代企业制度要求，完善法人治理结构。创新国有企业负责人管理体制，分类分层分步下放管理权限。

　　加强对国有资产的监管，健全监督体系，防止国有资产流失。加快国有经济布局和结构调整。以现代制造业、建筑业、商业、旅游服务业和基础设施等领域的改革为突破口，通过兼并、重组等方式做强一批大公司和大集团。尚未完成改制的国有企业，要加快规范的股份制改造。搞好104户企业的并购工作。运用拍卖、租赁、股份合作等多种方式，进一步放开搞活国有中小企业。认真做好首次经济普查工作。

　　(三)充分发挥奥运带动作用，提升城市现代化水平。以奥运建设全面启动为契机，从解决最为突出的矛盾入手，统筹城市规划、建设和管理，加快城市现代化建设步伐。

　　调整城市空间布局，努力提高城市规划水平。按照“两轴——两带——多中心”城市空间新格局，确保年内完成《北京市城市总体规划》修编，完善控制性详细规划。编制完成城市中心地区地下空间开发利用规划。按照“平原城市化、山区城镇化”方针，优先选择几个发展区位、产业基础和居住环境较好的卫星城，将其规划建设成各具特色的现代化新型城市，成为分流城区产业、人口和吸引农民进城的重要载体。

　　创新规划编制方法，吸收经济、社会等领域专家参与规划编制全过程。坚持规划的科学性、公开性、权威性。对一些重点地区和重要建设项目的规划设计，采取方案征集、招投标等方式确定；实施城市规划公示制度，强化规划管理。合理控制房地产开发规模和布局。

　　正确处理城市建设与古都风貌保护的关系，认真落实历史文化名城保护的各项规划、政策和措施，逐步做到保护有法可依、办事规范有序、资金保障有力。重点推进白塔寺、玉河等历史文化保护区的保护试点。切实加强世界文化遗产保护。全面实施《人文奥运文物保护计划》。

　　坚持“开放接轨、公开透明、节俭务实”筹办方针，切实搞好奥运场馆建设。认真贯彻“勤俭办奥运”，加大奥运场馆建设的组织协调力度，加强管理，确保安全、质量和工期。尽快完成尚未开工的新建和改扩建场馆的各项准备工作，力争早日开工。加紧进行奥林匹克公园中心区基础设施建设。

　　严格落实项目法人责任制，规范业主行为，加强对工程质量和安全生产的监督管理，认真实施国际奥委会和国家颁布的强制性质量标准和特殊要求，努力把奥运场馆建成一流水平的建筑精品。实行市场化、公开化的运行机制，推进“阳光工程”，实现“廉洁奥运”。

　　加快交通设施建设速度，逐步缓解城市交通拥堵。编制《北京交通发展纲要》。建管并重，综合治理，全面完成缓解交通拥堵第一阶段各项任务。把发展轨道交通放在首位，安全、优质、高效建设地铁四号线、五号线、十号线、奥运支线和机场线。加快市区南北通道建设，建成多条城市快速联络线和动物园、六里桥综合客运枢纽。

　　调动区县积极性，建设一批城市次干路和支路，加密城市路网，改善交通“微循环”。科学调整现有交通组织，完善交通管理设施，提高智能管理水平。坚持公交优先发展战略，完成南中轴路大容量快速公交试点。实施交通疏堵工程，重点改善望京、中关村等地区的交通状况，整顿崇文门路口等交通拥堵点段。继续治理机动车超限超载。加强停车规划和管理。加大交通宣传力度，提高市民交通文明意识。

　　围绕“绿色奥运”，进一步加大环境保护和生态建设力度。按照北京市生活垃圾治理白皮书确定的目标和任务，治理四环路到五环路之间垃圾堆积点，建成医疗废物和餐厨垃圾集中处理设施，推进生活垃圾减量化、资源化，使生活垃圾无害化处理率城近郊区达到93%、农村达到30%。加快城市污水治理，建成卢沟桥等污水处理厂及配套管线工程。

　　继续推进城近郊区公厕的改造和建设。加大固体废弃物、危险废弃物等污染防治力度。结合道路建设、文物修缮、拆除违法建设和农村环境治理，实施百项环境综合整治重点工程。抓好南北中轴线等地区的绿化美化，启动城市楔形绿地建设，巩固提高第一道绿化隔离地区的绿化水平，调整第二道绿化隔离地区的绿化用地规模，加快建设步伐。

　　大气污染治理进入攻坚阶段，奥运场馆及相关设施建设全面铺开，使治理难度加大。必须采取更加有效的措施，确保市区空气质量二级和好于二级的天数达到62%以上。

　　实行最严格的土地和水资源管理制度，建设节约型城市。坚决贯彻节约优先原则，把节地、节水和节能作为可持续发展的基本政策。严格保护基本农田，清理整顿各类开发区用地，进一步加大对土地违法问题的查处力度，依法处置闲置土地。实行土地垂直管理体制，规范控制建设用地协议出让范围，全面推行经营性土地使用权出让的招标、拍卖、挂牌制度。

　　建立水资源统一管理体制，组建水务管理部门，采取严格措施对水资源进行合理配置使用，保护好饮用水源。高质量建设南水北调北京段工程。制定节约用水管理办法和分阶段工作方案，实施阶梯水价，推动再生水回用和雨水利用，推广和更新节水器具，强化节水管理。开展节水宣传教育，提高市民节水意识。突出抓好节能工作，积极开发利用新能源，搞好资源的综合利用，努力发展循环经济。

　　深化城市管理体制综合改革。以增强各级政府社会管理和公共服务职能、强化社区民主自治功能为突破口，逐步实现政府职能到位、市场作用入位、社区功能归位。明确划分城市管理系统中各部门的职能、管理幅度与管理层次，建立健全城市公共管理和服务体系。发挥区县在城市管理中的职能和作用，强化对城乡结合部的综合治理。加强街道的统筹协调和综合管理能力，把城市管理的各项基础工作落在实处。

　　进一步搞好社区建设，完善社区功能，调动广大市民参与城市管理的积极性。做好对流动人口的服务和管理，改善外来务工人员的就业、子女就学和生活条件。稳定低生育水平，提高出生人口素质，把计划生育工作重心落实到社区。

　　各位代表，首都安全责任重于泰山，人民生命安全高于一切。我们要深刻汲取密云特别重大伤亡事故的惨痛教训，严格落实安全责任制，排查和消除各种重大事故隐患。建立城市统一的应急指挥系统，整合各类应急资源，完善各类突发事件应急预案，特别要切实解决预案落实和执行不到位的问题，提高防范和处置各类重大突发性事件的能力。

　　(四)关心群众切身利益，努力提高人民生活水平和质量。在全面建设小康社会的过程中，要不断提高城乡居民收入，不断满足人民群众的精神文化生活需要，更加重视人民群众的身体健康和生命安全，促进人的全面发展。

　　建立城乡一体化的就业服务体系，努力扩大就业规模。就业是民生之本。要着重发展第三产业和中小企业，注重发展个体、私营等非公有制经济，不断扩大就业容量。改善社区创业环境，再开发10万个社区就业岗位。完善市场导向的就业机制，建立城乡平等的就业制度，全面推行农村富余劳动力就业登记服务制度，将农转非人员纳入促进就业优惠政策范围，逐步实现农村富余劳动力与城镇劳动力在就业管理、培训和服务等方面平等对待。

　　高度重视再就业工作，认真落实各项优惠政策。抓好就业培训和指导，帮助失业人员转变就业观念，提高职业技能。组建创业指导中心，对失业人员创业提供“一站式”服务。建立公益性就业岗位定向分配制度，优先安置“4050”失业人员。街道开办的社区公益性就业组织对就业特困人员实行“托底”安置，确保安置率在95%以上。高度重视建设领域拖欠工程款和农民工工资问题，加大工作力度，为2005年基本解决这一问题打好基础。

　　建立城乡衔接的社会保障制度，保障城乡居民基本生活。依法扩大社会保障覆盖面。制定实施相关政策措施，解决当前反映比较突出的灵活就业人员和外来劳动力参保问题。继续推进基本医疗保险制度改革，采取措施减轻参保人员的医疗费用负担。完善事业单位参加养老保险政策，制定实施《北京市生育保险规定》。

　　大力推进补充保险制度建设，鼓励有条件的企业建立补充养老保险，进一步落实和规范企业补充医疗保险，提倡个人参加商业保险，形成多层次的保险模式，提高保障水平。积极探索建立以区县为统筹单位的农村社会保障体系，重点解决失地农民的就业和社会保障问题。进一步完善以大病统筹为主的新型农村合作医疗制度，扩大试点范围。

　　加快农村养老保险制度改革步伐，研究城镇与农村养老保险制度衔接办法。强化社会保险基金征缴和管理，确保基金安全。构建新型城乡社会救助体系。制定不同低保家庭的分类救助办法和家庭收入核实办法，确保符合条件的家庭进入保障范围。完善医疗、教育、住房等专项和临时救助政策，解决低收入家庭的特殊性、突发性困难。切实保障妇女、未成年人、老年人、残疾人的合法权益，在全社会形成尊老助残的氛围。

　　合理安排经济适用住房建设规模和进度，确定适当户型标准，实行购买申请审批公示制度，加强上市交易管理。扩大廉租住房覆盖面，解决好“双低收入”家庭的居住困难。注意处理好各种关系，基本解决标准租私房问题。坚决打击违法拆迁行为，依法实施文明拆迁。强化政府公共卫生管理职能，提高群众健康水平。

　　以建立健全城乡一体的公共卫生体系和突发公共卫生事件应急机制为重点，提升服务能力和水平。确保卫生事业投入有明显增长，将资金重点用于突发事件应急、农村卫生服务、城市基本医疗服务等领域。建设北京公共卫生应急指挥中心和紧急医疗救援中心，完善信息网络。高度重视农村卫生工作，加大对山区、半山区、偏远贫困地区的投入力度，把乡镇卫生院、村卫生室纳入公共卫生体系，重点加强建设，落实城市卫生资源支农措施。

　　完善预防、治疗、保健等六位一体的社区卫生服务。整合优化现有医疗资源配置，加快办医主体多元化步伐。发挥中西医结合优势。加强爱国卫生工作，大力普及卫生常识和健康教育。落实《北京防治传染性非典型肺炎应急预案》，切实有效地防止疫情反复。依靠科学，依靠法律，依靠群众，严密防控高致病性禽流感疫情，全力以赴打赢这场阻击战。

　　推进教育文化体育事业发展，不断满足人们日益增长的文化需求。全面推进素质教育，调整教育结构，提高教育质量，统筹城乡、区域和各级各类教育协调发展。将义务教育特别是农村义务教育作为重中之重，加大对困难区县和农村地区的投入力度。颁布实施新的《中小学办学条件标准》，重点改善半山区和农村平原地区中小学的教学设施和校园环境。加快推进城区初中校建设工程，基本完成办学相对困难高中校的改造。

　　与经济社会发展紧密结合、与人才需求结构相适应，加快高等教育创新，增强学生的就业、创新和创业能力。大力发展城乡职业教育、成人教育，加强各类技能教育培训。深化教育体制改革，积极发展多种形式办学，提高民办教育办学规模和水平，支持引进外国优质教育资源。强化师资队伍建设，努力提高整体素质。继续加大治理教育乱收费的力度。

　　加快推进文化体制改革试点工作，按照扶持一批、转企一批、重组一批、剥离一批的原则，对文化企事业单位进行分类改革。以街道、乡镇文化中心和社区、村文化室为重点，继续推进基层文化建设。建成首都博物馆、北京天文馆新馆等文化设施。广泛开展全民健身活动，办好亚洲杯足球赛等重大国际体育赛事，出色完成雅典奥运会火炬在京传递和奥运接旗活动，实施奥运夺金计划，推进国际化体育中心城市建设。

　　各位代表，办好直接关系群众生活的重要实事，是市政府解决群众实际问题的有效途径和手段。今年，确定了56件实事，我们将明确责任，保证投入，确保年底兑现。

　　(五)加强社会主义精神文明建设和民主法制建设，维护首都改革发展稳定大局。以建设社会主义政治文明和精神文明的首善之区为目标，扎实推进首都精神文明建设，坚持和完善社会主义民主制度，提高依法治市水平，巩固和发展首都奋发向上、社会和谐、安定团结的政治局面。

　　切实加强社会主义精神文明建设。继续深入开展“三个代表”重要思想学习教育活动，以邓小平理论和“三个代表”重要思想武装干部群众，努力使科学理论成为全市人民团结奋斗的共同思想基础和精神支柱。以迎接新中国成立55周年为契机，以“建设新北京、办好新奥运”为主题，深入开展热爱祖国、热爱首都的群众性教育活动，培育和弘扬民族精神，强化广大市民的首都意识、法律意识和公德意识，切实提高市民文明素质。

　　广泛普及奥运知识，继续开展市民讲外语活动。加强哲学社会科学研究和科普宣传教育，大力弘扬科学精神。深化国防教育，广泛开展“双拥”共建活动，加强国防后备力量建设，支持驻京部队后勤保障社会化。深入开展丰富多彩的群众性精神文明创建活动，全面推进文明社区、文明村镇、文明行业等建设，逐步形成良好的社会风气。

　　坚持完善社会主义民主制度。坚决贯彻人大及其常委会决议，自觉接受监督。坚持重大事项报告制度，根据市人大常委会的要求，准备好政府有关工作报告，接受审议。市政府组成人员要认真做好述职，虚心听取评议，及时加以整改。主动加强与政协及各民主党派、工商联和无党派人士的联系，在政府决策过程中广泛听取各界意见，认真研究采纳政协常委会和各民主党派建议案，接受民主监督。

　　增强责任感和主动性，严肃负责地办理人大议案、建议和政协提案，提高质量和效率。全面落实党和国家的民族、宗教政策，切实做好侨务和对台工作。巩固和扩大基层民主，完善居民自治和村民自治，深化厂务公开，保障人民群众依法直接行使民主权利。

　　全面推进依法行政。认真落实《北京市依法治市工作规划》。增强依法行政意识，提高政府的法治化管理水平。坚决执行《行政许可法》，按期完成行政许可事项、规定和实施主体的清理工作，严格按照法定权限和程序行使权力、履行职责，加快建立权责明确、行为规范、监督有效、保障有力的执法体制。加强行政执法队伍建设，端正执法思想，牢固树立执法为民观念，实施规范化管理和监督。

　　建立行政投诉受理中心，坚决查处公务人员的行政违法行为。建立和完善重大问题集体决策制度、专家咨询制度、社会公示制度和听证制度，以及决策责任制度，不断提高决策的民主化、科学化水平。健全教育、制度、监督并重的惩治和预防腐败体系，扎实推进廉政建设。大力弘扬求真务实精神，坚持正确的政绩观，脚踏实地，埋头苦干，切实做到“为民、务实、清廉”。

　　全力维护首都社会稳定。稳定是改革和发展的前提。要把改革的力度、发展的速度与社会可承受程度统一起来，把不断提高人民生活水平作为处理改革发展稳定关系的重要结合点，坚决维护首都改革发展稳定的大局。要以维护人民利益为根本出发点和落脚点，在各项决策及日常工作中高度重视并切实维护群众利益，认真解决群众最关心、最迫切需要解决的实际问题。

　　努力做好人民来信来访和人民内部矛盾纠纷排查调处工作，力争把矛盾解决在基层，化解在萌芽状态。严密防范和坚决打击国际国内敌对势力的渗透、颠覆、分裂等破坏活动，坚持不懈地开展同“法轮功”等邪教组织的斗争。巩固和发展严打整治斗争成果，建设社会治安防控体系，切实维护首都的安全稳定。

　　各位代表，历史的经验证明，任何风险和挑战都无法阻挡首都现代化建设的前进步伐，任何困难和挫折都无法改变首都人民创造幸福生活的美好愿望。展望未来，我们充满信心。

　　让我们紧密团结在以胡锦涛同志为总书记的党中央周围，高举邓小平理论伟大旗帜，全面贯彻“三个代表”重要思想，认真落实党的十六大、十六届三中全会和市第九次党代会精神，与时俱进，开拓创新，扎实工作，为实现“新北京、新奥运”战略构想，为率先基本实现现代化而努力奋斗！
